# Research progress on the relationship between *Helicobacter pylori* infection and iron deficiency anemia

**DOI:** 10.3389/fmicb.2025.1552630

**Published:** 2025-03-25

**Authors:** Sugui Pu, Ze Zhuang, Na Liu, Qian Luo, Dekui Zhang

**Affiliations:** ^1^Department of Gastroenterology, The Second Clinical Medical College of Lanzhou University, Lanzhou University Second Hospital, Lanzhou, China; ^2^Key Laboratory of Digestive Diseases, Lanzhou University Second Hospital, Lanzhou, China

**Keywords:** *Helicobacter pylori*, eradication therapy, Iron deficiency anemia, hemoglobin, review

## Abstract

*Helicobacter pylori* (*H. pylori*) infection affects around half of the global population and is a globally highly prevalent pathogen that is closely linked not only to gastrointestinal diseases such as chronic atrophic gastritis, functional dyspepsia and peptic ulcer but also to the development and progression of a variety of extra-gastrointestinal diseases. Numerous studies have shown the correlation between *H. pylori* infection and iron-deficiency anemia (IDA). The prevalence of *H. pylori* infection is higher in individuals with IDA, and the hemoglobin level of patients with IDA can be increased to different degrees or even returned to normal following active *H. pylori* eradication. However, this conclusion is still controversial. In this paper, a comprehensive literature search was conducted using the PubMed/MEDLINE/Web of Science database, combining the following terms: “*Helicobacter pylori*,” “*Helicobacter pylori* infection,” “iron deficiency anemia,” “iron deficiency,” “iron absorption,” “iron malabsorption,” “serum iron,” “hemoglobin,” “pathogenesis,” “mechanism,” and “eradication therapy.” Through extensive literature searches, the correlation between *H. pylori* infection and IDA, its potential mechanism, and the efficacy of *H. pylori* eradication therapy in IDA patients have been comprehensively discussed. We conclude that the majority of existing studies have confirmed the correlation between *H. pylori* infection and IDA, indicating that patients with *H. pylori* infection are more likely to develop IDA and that the prevalence of *H. pylori* infection is higher in individuals with IDA. Compared with iron supplementation alone, combining *H. pylori* eradication with iron supplementation is more effective in treating IDA, particularly in unexplained or refractory IDA cases. These findings provide valuable insights for clinicians managing patients with unexplained or refractory IDA.

## 1 Introduction

*Helicobacter pylori* (*H. pylori*) is a spiral microaerobic bacterium capable of surviving in strongly acidic environments, often colonizing the epithelium of the human gastric and duodenal mucosa (Zeng et al., 2015). It is a common culprit in many gastrointestinal diseases, such as chronic atrophic gastritis (Yang et al., 2021), functional dyspepsia (Chey et al., 2017) and peptic ulcer (Sugano and Howden, 2021). Furthermore, a growing body of research has linked *H. pylori* to a variety of extra-gastrointestinal diseases, including coronary heart disease (Sun et al., 2023), atrial fibrillation (Wang et al., 2015), bronchial asthma (Miftahussurur et al., 2017; Chen and Blaser, 2008; Elias et al., 2020), Parkinson’s (Wei et al., 2024), non-alcoholic fatty liver disease (Abdel-Razik et al., 2018; Kountouras et al., 2024), and many others. It may play a role in the generation and progression of these diseases. Iron deficiency anemia (IDA) is one of the most prevalent micronutrient deficiencies globally (Stoltzfus, 2001), affecting 30% of the population worldwide and more than half of children in developing countries (DeMaeyer and Adiels-Tegman, 1985). The correlation between *H. pylori* infection and IDA has attracted considerable attention in the past few years. A variety of investigations have shown that the rate of *H. pylori* infection is higher in individuals with IDA, and the hemoglobin level of patients with IDA can be increased to different degrees or even returned to normal following active *H. pylori* eradication, but this conclusion is still controversial. This paper reviews the correlation between *H. pylori* infection and IDA, its underlying mechanisms, and the efficacy of *H. pylori* eradication in patients with IDA, particularly those with unexplained or refractory IDA.

## 2 Epidemiological characteristics of *Helicobacter pylori* and IDA

*H. pylori* infection is an important public health challenge worldwide. It is particularly prevalent in developing countries, affecting about 50% of the world’s population. The prevalence of *H. pylori* infection exhibits significant geographic variation worldwide. According to statistics, the prevalence in Northern Europe and North America is relatively low, affecting approximately 33.3% of the adult population. In contrast, prevalence rates exceed 50% of the population in Southern Europe, Eastern Europe, South America, and several Asian regions (Eusebi et al., 2014). China, one of Asia’s most populous countries, has an *H. pylori* infection rate of 44.2%, with an estimated 589 million Chinese infected (Ren et al., 2022). Humans are the only host and main source of *H. pylori* infection (Coelho et al., 2018). It is mainly transmitted through the fecal-oral, oral-oral, and gastro-oral routes and is more commonly transmitted within the family, especially from mother to child (Burucoa and Axon, 2017). In an Iranian study (Mamishi et al., 2016), fecal and blood samples from urea breath test (UBT)-positive children and their parents were tested by fecal antigen detection assay and enzyme-linked immunosorbent assay, and RAPD (randomly amplified polymorphic DNA) fingerprints of genomic DNA were obtained from *H. pylori* isolates from all subjects. This study showed that the primary mode of intrafamily *H. pylori* transmission in Iran is from mother to child. Overall, the prevalence of *H. pylori* infection was positively correlated with age (Ozturk et al., 2021; Zhestkova et al., 2018; Forman et al., 1993; Xie et al., 2024), which may be related to changes in the gastric environment with age. Compared to younger individuals, older adults exhibit reduced gastric acid secretion (Kinoshita et al., 1997; Blackman et al., 1970; Grossman et al., 1963; Baron, 1963), delayed gastric emptying (Orr and Chen, 2002), and decreased microbial diversity in the stomach (Liatsos et al., 2022; Shin et al., 2020). In addition, its prevalence varied significantly by geographic region, race, ethnicity, economic level, and social status (Bautista et al., 2015; Nguyen et al., 2015; Xu et al., 2017). With socio-economic development as well as improvement and enhancement of human living standards, *H. pylori* infection rates have declined globally. However, *H. pylori* prevalence generally remains high in developing countries (Zhou et al., 2022).

IDA is a form of anemia caused by insufficient intake, impaired absorption, and excessive loss of iron in the human body (Hershko and Skikne, 2009). It is clinically characterized by a constellation of symptoms, including generalized weakness, dizziness, palpitations, chest tightness, and drowsiness (Auerbach and Adamson, 2016). It has a significant impact on human health, as well as on social and economic development (McLean et al., 2009).

IDA has been recognized as the most prevalent nutritional deficiency globally, with approximately 30% of the population suffering from this disease (Kumar et al., 2022). The prevalence rate varies significantly among countries with different development levels: 9.1% in high-development countries, 25.7% in middle-development countries, and 42.8% in low-development countries (McLean et al., 2009). In addition, there is a considerable overlap between areas with a high incidence of IDA and regions with a high prevalence of *H. pylori* infection (Burns et al., 2017). While IDA more commonly impacts children and women of childbearing age, adult males can also be at risk depending on their socio-economic and health conditions (Bathla and Arora, 2022; Kassebaum et al., 2014).

## 3 Association of *Helicobacter pylori* infection with IDA or low serum iron

The possibility that *H. pylori* infection may result in IDA was first noted in 1991 when a Belgian case report indicated that the hemoglobin value of an adolescent girl with IDA returned to normal, and her anemia-related symptoms completely disappeared after *H. pylori* eradication alone, without any iron supplementation (Blecker et al., 1991). Since then, many studies and meta-analyses have described the correlation between *H. pylori* infection and IDA, but some studies have failed to find a causal relationship. Overall, *H. pylori* testing and eradication therapy are recommended for patients with unexplained IDA (Malfertheiner et al., 2012).

Firstly, numerous studies have indicated an increased susceptibility to IDA in individuals with *H. pylori* infection. A meta-analysis conducted by Hudak et al. in 2017 showed that people with evidence of *H. pylori* infection had 1.72 times the risk of developing IDA compared to uninfected people (Hudak et al., 2017). Another meta-analysis (Muhsen and Cohen, 2008) also showed that *H. pylori* infection was a risk factor for reduced iron stores in the body and that infected individuals were more likely to develop IDA; pooled odds ratio (OR) 2.8 (95% CI 1.9–4.2, *p* < 0.001). A retrospective analysis conducted by Xu et al. showed that after adjusting for baseline information and confounders, the prevalence of anemia was notably higher in patients in the *H. pylori* (+) group than in the *H. pylori* (−) group (Xu et al., 2017). Infected patients were 1.39 times more likely to have moderate-to-severe anemia and 1.05 times more likely to have mild anemia than uninfected patients (Xu et al., 2017). Miernyk et al. also mentioned that most *H. pylori*-infected individuals suffered from IDA compared to uninfected individuals and that *H. pylori* eradication increased serum ferritin levels (Miernyk et al., 2013).

Secondly, some researchers have reported a higher rate of *H. pylori* infection in IDA patients. Demerdash et al. performed a case–controlled study consisting of 104 cases of unexplained or refractory IDA and 70 healthy controls, which revealed that *H. pylori* infection was more prevalent in unexplained or refractory IDA patients (61.5%), much higher than in healthy controls (14.3%), and statistically significant (*p* < 0.001) (Demerdash et al., 2018). Another cross-sectional study from Saudi Arabia showed that the prevalence of *H. pylori* infection in patients with IDA was 62% and that *H. pylori* infection was associated with unexplained IDA (Nasif et al., 2021).

Furthermore, some studies indicate that *H. pylori* infection can cause reduced serum iron or ferritin levels (Kishore et al., 2021; Lee et al., 2022; Berg et al., 2001; Parkinson et al., 2000; Cardenas et al., 2006; Mwafy and Afana, 2018). For example, Mwafy et al. showed that serum iron and hemoglobin levels are significantly lower in *H. pylori*-positive patients and that *H. pylori* may be the causative agent of IDA formation (Mwafy and Afana, 2018).

However, the correlation between *H. pylori* infection and IDA has been controversial. Some studies have failed to find a causal relationship. American scholars Tseng et al. followed up on 508 adult patients diagnosed with *H. pylori* infection and IDA for 2 years, and they observed that the anemia resolved in most patients with or without *H. pylori* treatment, there was no specific evidence linking *H. pylori* infection to IDA (Tseng et al., 2019). Similarly, a study by John et al. showed no significant correlation between unexplained IDA or iron deficiency (ID) and *H. pylori* infection in an elderly population (John et al., 2018). An Iranian study of school-age children also showed no statistical distinction in the proportion of IDA between the *H. pylori* positive and negative groups (Zahmatkeshan et al., 2019). In addition, several studies conducted in Brazil (Araf et al., 2010; Alvarenga et al., 2010), Korea (Choi, 2003), Sweden (Sandström et al., 2014), Egypt (El-Said et al., 2017), and Iran (Zamani et al., 2011) were unsuccessful in finding any notable correlation between *H. pylori* infection and IDA.

It is important to point out that most of the current data supporting the association between *H. pylori* infection and IDA are primarily from clinical trials carried out on children and premenopausal women who have higher iron needs, as well as those living in areas where *H. pylori* is highly endemic. The discrepancies in the outcomes may be attributed to variations in the geographic and ethnic distribution of participants, inclusion and exclusion criteria, sample sizes, anemia detection techniques, and *H. pylori* infection detection methods across the studies. Additionally, the majority of current research on this topic are cross-sectional and retrospective analyses, lacking randomized controlled trials and other prospective studies.

## 4 Potential mechanisms of *Helicobacter pylori* infection associated with ID or IDA

The mechanisms by which *H. pylori* infection leads to ID or IDA are not fully understood, and current researches provide the following explanations:

### 4.1 Reduce gastric acid and ascorbic acid levels, which in turn affect iron absorption

Dietary iron absorption requires normal concentrations of gastric acid and ascorbic acid (AA) (Betesh et al., 2015; Silva and Faustino, 2015). Both elevated gastric pH and reduced AA levels reduce the reduction of dietary Fe3+ to Fe2+, thereby preventing the absorption of non-heme iron (Annibale et al., 2003). *H. pylori* infections (Sarker et al., 2012; Harris et al., 2013) and atrophic gastritis caused by its persistent inflammatory response (Annibale et al., 2020; Kishikawa et al., 2020) can reduce gastric acid secretion. AA is considered to be the most effective enhancer of iron absorption (Conrad et al., 1999; Rathbone et al., 1989). It not only reduces trivalent iron to the ferrous form, which maintains its solubility in the alkaline environment of the duodenum, but also forms a chelate with ferric chloride in the acidic environment of the stomach, and this complex is also stable at pH > 3 (Bothwell et al., 1989). However, AA is very unstable at elevated pH, and *H. pylori* infection also reduces its bioavailability and accelerates its degradation, which in turn reduces iron absorption and causes iron deficiency anemia (Mei and Tu, 2018).

### 4.2 By competing with the host for iron uptake resulting in IDA

*H. pylori* competes with the host for iron uptake through certain outer membrane proteins it possesses. It can affect host cell polarity locally. Through the synergistic action of its virulence factors, cytotoxin-associated gene A (CagA) and vacuolar cytotoxin A (VacA), *H. pylori* mislocalizes transferrin and transferrin receptor from the basolateral side of intestinal epithelial cells via cytoplasmic translocation pathway to the apical cell surfaces of the bacterial microcolony growth sites, resulting in an increased release of transferrin from the apical side of the cells, and facilitating the utilization of the iron contained in the holo-transferrin. The polarized epithelium is also used as a “filter” to protect itself from potential host toxicity defense molecules (Tan et al., 2011). Competition with the host for iron absorption further promotes *H. pylori* growth and gastrointestinal colonization. Flores et al. used knockout strains to elucidate that the *Helicobacter pylori* virulence factor CagA increases iron uptake and lysosomally unstable iron storage in infected adenocarcinoma gastric cell line (AGS) cells, a gastric epithelial cell line commonly utilized to simulate *in vitro H. pylori* infection, thereby interfering with intracellular iron homeostasis in AGS cells (Flores et al., 2017). Furthermore, the expression of iron-inhibited outer membrane proteins (IROMPs) involved in iron acquisition is enhanced under iron deficiency conditions in specific *H. pylori* strains infecting IDA patients, suggesting that IDA strains may utilize large amounts of iron from the gastric mucosa, thereby promoting the development of IDA in iron-deficient hosts (Lee et al., 2009).

### 4.3 Influence iron regulation mechanism in the human body by upregulating hepcidin level

*H. pylori* infection can contribute to ID by influencing iron absorption by enterocytes and iron release by macrophages through the upregulation of hepcidin levels (Mendoza et al., 2019). Hepcidin, a hormone consisting of 25 amino acids produced mainly by hepatocytes, is a central regulator of iron metabolism in the body and prevents iron absorption in the small intestine (Nicolas et al., 2001). Ferroportin-1 (Fpn1) is the only known mammalian iron exporter and mediates the exocytosis of intracellular Fe2+ (Le and Richardson, 2002). Hepcidin irreversibly binds to Fpn1 on the surface of enterocytes and macrophages via the hepcidin/ferroportin-1 system, inducing its internalization and subsequent lysosomal degradation, thereby reducing iron escape (Silva and Faustino, 2015). Hepcidin has also been associated with reduced transcription of the gene encoding divalent metal transporter 1 (DTM1), a transmembrane iron importer, which utilizes the proton gradient existing between the gut lumen and the enterocyte cytoplasm to accomplish the exchange of Fe2+ and H+, thus participating in the absorption of dietary iron (Gunshin et al., 1997). Hepcidin gene (HAMP) expression is upregulated during high iron levels, inflammation and infection, while hypoxia, anemia and erythropoiesis inhibit its expression (Silva and Faustino, 2015). The A component of *H. pylori* lipopolysaccharide stimulates increased production of cytokines interleukin-6 (IL-6) and interleukin-1beta (IL-1beta), which in turn stimulates hepcidin production in hepatocytes, leading to reduced iron uptake and mobilization from liver and macrophage deposits, impeding iron absorption and release (Pellicano and Rizzetto, 2004; Cherian et al., 2008; Casals-Pascual et al., 2012; Freire de Melo et al., 2012). Sapmaz et al. indicated that the level of hepcidin secretion was increased in patients with *H. pylori* infection, and the level of hepcidin *in vivo* returned to normal after eradication of *H. pylori*, suggesting that the increased level of hepcidin is associated with *H. pylori* infection (Sapmaz et al., 2016). Mendoza et al. found that *H. pylori* infection increased synthesis of hepcidin in children and that *H. pylori* infection showed a correlation with IDA in children with higher levels of hepcidin, whereas this correlation was not statistically significant in children with lower levels of hepcidin, indicating that *H. pylori* contributes to the development of IDA by promoting hepcidin synthesis (Mendoza et al., 2019).

### 4.4 *Helicobacter pylori* gene polymorphism is associated with IDA

Japanese scholars Yokota et al. determined the nucleotide sequences of *napA*, *fur* and *feoB* involved in iron ion uptake in 24 *H. pylori* strains from IDA patients (IDA strains) and 25 *H. pylori* strains from *H. pylori* gastritis patients without anemia (non-IDA strains), and showed that the frequency of the neutrophil-activating protein A (NapA) encoded by napA, with threonine at amino acid residue No. 70(thr70 type NapA) was notably higher in IDA strains compared to non-IDA strains. *H. pylori* carrying thr70-type NapA has a strong iron uptake capacity, which is implicated in the pathogenesis of IDA (Yokota et al., 2013). Kato et al. found that by comparing the bacterial genome-wide expression profiles of *H. pylori*-infected children with and without IDA, the expression levels of 29 genes were significantly higher, and 11 genes were significantly lower in children with IDA. Among them, the high expression of the sialic acid binding adhesin (*SabA*) gene is important in causing IDA, especially for children with increased daily iron requirements (Kato et al., 2017). Moreover, VacA may act synergistically with *SabA* in the development of IDA. In addition, tumor necrosis factor-alpha (TNF-alpha), a pro-inflammatory cytokine that may contribute to IDA, can also be upregulated by *H. pylori* (Gravina et al., 2020).

### 4.5 Increase the risk of overt and covert blood loss leading to IDA

*H. pylori* infection can cause gastrointestinal mucosal lesions, increasing the risk of overt and occult blood loss. However, most published cases of IDA associated with *H. pylori* did not reveal hemorrhagic lesions on endoscopy and had negative fecal occult blood tests. Therefore, gastrointestinal mucosal bleeding may not be the main cause of IDA due to *H. pylori* infection.

## 5 Efficacy of *Helicobacter pylori* eradication in IDA, especially unexplained or refractory IDA

IDA is among the limited number of extra gastric diseases for which *H. pylori* eradication is explicitly advised by the Maastricht VI/Florence guidelines and the IDA guidelines (Malfertheiner et al., 2017). The strongest evidence for a causal relationship is the cure of anemia by *H. pylori* eradication (Hershko and Camaschella, 2014). Many studies have confirmed the benefit of *H. pylori* eradication in the treatment of IDA, especially in unexplained IDA or refractory IDA [“unexplained IDA” is defined as IDA for which gastrointestinal endoscopy cannot identify the reason, whereas “refractory IDA” applies when a significant proportion of patients do not respond to iron supplementation of at least 100 mg per day for 4–6 weeks (Hershko and Camaschella, 2014)]. However, several studies negate this effect.

Firstly, many studies and meta-analyses (Hudak et al., 2017; Huang et al., 2010) have indicated that *H. pylori* eradication therapy plus iron supplementation is a more effective strategy for improving IDA than iron supplementation alone. A meta-analysis of 16 randomized controlled trials (RCTs) involving 956 patients showed that anti- *H. pylori* treatment together with iron therapy resulted in a statistically significantly higher incremental increase in hemoglobin, serum iron, and serum ferritin from baseline to endpoint than iron therapy alone. Moreover, this effect was more pronounced in patients with moderate to severe anemia (Yuan et al., 2010). Demerdash et al. randomized 64 *H. pylori*-infected patients with unexplained or refractory IDA into two groups: Group A was treated with *H. pylori* eradication plus iron therapy, and Group B was treated with iron therapy alone. After 3 months of treatment, hemoglobin level, mean erythrocyte volume (MCV), mean corpuscular hemoglobin (MCH), serum iron and ferritin levels were significantly improved in subjects in group A (all *p* < 0.001), whereas the difference between the levels of these parameters in group B before and after treatment was not statistically significant. This research indicates that *H. pylori* eradication therapy plus iron supplementation are more beneficial in treating IDA (Demerdash et al., 2018). Similarly, a survey by Hudak et al. supported this finding (Hudak et al., 2017). In addition, a Mexican study showed that *H. pylori* eradication plus iron supplementation was beneficial in increasing functional iron stores in children compared to uninfected children who were supplemented with iron (Duque et al., 2010).

More importantly, there is no shortage of studies showing that in some patients, even *H. pylori* eradication alone without iron supplementation can correct IDA. A prospective study followed 44 male patients with unexplained or no clear source of bleeding IDA for 4 ~ 69 months, most of whom had a poor initial response to oral iron therapy, but after eradication of *H. pylori*, all participants returned to normal hemoglobin levels, and 4 of them returned to normal hemoglobin in the absence of oral iron after *H. pylori* eradication, providing strong evidence for causality between *H. pylori* infection and IDA (Hershko et al., 2007). Results from another before-and-after observational study showed that *H. pylori* eradication reduced weight loss and prevented subclinical IDA in older adults (Maruyama et al., 2017). A survey by Miernyk et al. demonstrated that low serum iron levels are linked to *H. pylori* infection and that eradication alone, without iron supplementation, was effective in overcoming anemia and improving patients’ quality of life (Miernyk et al., 2013). A double-blind, randomized trial in the United States of children with non-iron deficient, asymptomatic *H. pylori* infection found that the mean change in serum ferritin from baseline was increased threefold in children eradicated from *H. pylori* compared with those who remained infected (Cardenas et al., 2011). Tanous et al. retrospectively analyzed the medical records of 60 children with endoscopically diagnosed *H. pylori* infection. They showed that successful eradication contributed to the improvement of iron status in children with refractory IDA (Tanous et al., 2022). Besides, the results of an RCT showed a significant increase in hemoglobin, erythrocyte pressure volume and MCV in anemic *H. pylori*-positive patients after eradication but no significant difference in serum ferritin levels (Emiralioglu et al., 2015).

All the above studies provide evidence that *H. pylori* eradication is beneficial in the therapy of IDA, especially unexplained IDA in children. In contrast, some studies have shown little or no change in IDA-related hematological parameters after *H. pylori* eradication. A prospective study followed 508 adult patients with unexplained IDA and *H. pylori* infection. After 2 years of observation, the difference in hemoglobin levels was not statistically significant between subjects who did or did not receive eradication therapy (Tseng et al., 2019). In Bangladesh, where *H. pylori* is highly endemic, a study of 200 children found that *H. pylori* was neither the cause of IDA/ID nor the failure of iron supplementation therapy (Sarker et al., 2008). Besides, a study in Saudi Arabia of schoolchildren infected with *H. pylori* reported that anti- *H. pylori* treatment did not significantly improve serum ferritin levels without iron supplementation (Ali Habib et al., 2013).

## 6 Characteristics of the correlation between *Helicobacter pylori* infection and IDA in children

Due to the characteristics of children’s growth and development, they are more susceptible to developing IDA associated with *H. pylori* infection than adults (Seo et al., 2002). Moreover, *H. pylori* infection predominantly occurs during childhood (Suerbaum and Michetti, 2002). Therefore, it is essential to separately examine the relationship between *H. pylori* infection and IDA in children.

First, the effects of *H. pylori* infection on iron metabolism in children align with those described in Section 4 of this article. However, it is important to note that children with IDA, particularly those under 2 years of age or exclusively breastfed, often exhibit low or deficient vitamin D levels (Yoon et al., 2012). Low vitamin D levels reduce the inhibitory effect on the transcription of the HAMP gene, leading to increased hepcidin expression and impaired iron absorption and utilization (Bacchetta et al., 2014). Consequently, in treating children with IDA and *H. pylori* infection, it is crucial to assess their vitamin D levels and provide supplementation if necessary. Additionally, during periods of rapid growth in children, the increased expression of the *H. pylori* Sab gene significantly contributes to the development of IDA (Kato et al., 2017). Thus, children with both *H. pylori* infection and IDA should actively undergo eradication therapy.

Second, unlike adult IDA patients, children with IDA and iron deficiency (ID) experience significant adverse effects on growth, development, and cognitive function (Barks et al., 2021; McCann et al., 2020; Cusick et al., 2018; Lozoff, 2011). As a result, *H. pylori* eradication therapy is particularly critical for children with refractory IDA. The Joint ESPGHAN/NASPGHAN Guidelines for the Management of *H. pylori* in Children and Adolescents (Update 2016) also recommend testing for and treating *H. pylori* infection in children with refractory IDA after excluding other potential causes (Jones et al., 2017).

## 7 Discussion

From the above, it can be seen that although it is still controversial whether *H. pylori* infection is associated with IDA, most of the available studies are positive about this, and many of them have confirmed that *H. pylori* eradication plus conventional iron supplementation therapy is more rapid and effective in the treatment of IDA than iron supplementation therapy alone, especially in unexplained or refractory IDA, which is considered to be strong evidence to argue for a causal relationship between *H. pylori* infection and IDA. Several guidelines have now recommended eradication therapy for *H. pylori*-positive IDA patients. Concerning the association mechanism, some current studies provide explanations but lack strong evidence, and some scholars believe that hepcidin occupies an important position in the relationship between them. Therefore, further in-depth exploration of the correlation between *H. pylori* infection and IDA, the mechanism, and the efficacy of *H. pylori* eradication therapy for IDA is needed, which will bring some hope to IDA patients, especially those with unexplained IDA or refractory IDA. It should be noted that the majority of current studies investigating the association between *H. pylori* infection and IDA are retrospective and observational designs. Future research should prioritize well-designed prospective RCTs and experimental studies to establish a more robust evidence base and elucidate the underlying pathophysiological mechanisms.

In addition, our review has several limitations. First, this study is a narrative review, which lacks systematic literature screening and quality assessment criteria compared to a systematic review. This approach is more susceptible to researchers’ subjective biases, potentially compromising the objectivity and reliability of the conclusions. To address this limitation, we developed a comprehensive literature search strategy, utilizing multiple databases and search methods to minimize the risk of omitting significant studies. Furthermore, all authors independently participated in the literature screening process to enhance the objectivity of the selection. Second, the prevalence of *H. pylori* infection and IDA varies significantly across different populations (Bautista et al., 2015; Nguyen et al., 2015; Xu et al., 2017; McLean et al., 2009), with children and pregnant women being particularly vulnerable to IDA (Bathla and Arora, 2022; Kassebaum et al., 2014). However, our review did not analyze the data based on specific population characteristics, which limits the generalizability of our findings. Future studies should further investigate the association between *H. pylori* and IDA in diverse populations to provide more targeted insights.

## 8 Conclusion

So far, the majority of existing studies have confirmed the correlation between *H. pylori* infection and IDA, indicating that patients with *H. pylori* infection are more likely to develop IDA and that the prevalence of *H. pylori* infection is higher in individuals with IDA. Compared with iron supplementation alone, combining *H. pylori* eradication with iron supplementation is more effective in treating IDA, particularly in unexplained or refractory IDA cases. These findings provide valuable insights for clinicians managing patients with unexplained or refractory IDA.
